# HTLV-1-associated diseases: a systematic review

**DOI:** 10.1590/S1678-9946202668054

**Published:** 2026-08-07

**Authors:** Rosa Maria Nascimento Marcusso, Víctor Ângelo Folgosi, Augusto César Penalva de Oliveira, Jorge Casseb

**Affiliations:** 1 Instituto de Infectologia Emilio Ribas, São Paulo, São Paulo, Brazil; 2 Universidade de São Paulo, Faculdade de Medicina, Divisão de Dermatologia, Laboratório de Investigação Médica (LIM-56), São Paulo, São Paulo, Brazil; 3 Universidade de São Paulo, Faculdade de Medicina, Instituto de Medicina Tropical de São Paulo, São Paulo, São Paulo, Brazil

**Keywords:** HTLV-1, HAM/TSP, Adult T-cell Leukemia/Lymphoma, Proviral load, Inflammatory diseases.

## Abstract

Human T-cell lymphotropic virus type 1 (HTLV-1) is a human retrovirus associated with a broad spectrum of clinical manifestations. While most people living with HTLV-1 remain asymptomatic, a proportion develops severe conditions such as adult T-cell leukemia/lymphoma or HTLV-1-associated myelopathy/tropical spastic paraparesis. Increasing evidence also indicates that HTLV-1 infection is related to multiple systemic complications involving different organ systems (including neurological, pulmonary, dermatological, rheumatological, and ophthalmological disorders) and opportunistic infections. Given the growing recognition of these diverse manifestations, this review aims to compile and synthesize the scientific evidence regarding clinical manifestations, co-infections, opportunistic diseases, and complications related to HTLV-1 infection.

## INTRODUCTION

Human T-cell lymphotropic virus type 1 (HTLV-1) infection is associated with a broad spectrum of clinical manifestations affecting multiple organ systems^1^ ^-^ ^4^. Although many people living with HTLV-1 (PLWHTLV-1) remain asymptomatic, persistent viral infection can lead to severe diseases, particularly adult T-cell leukemia/lymphoma (ATL) and HTLV-1-associated myelopathy/tropical spastic paraparesis (HAM/TSP)^5^ ^),(^ ^6^. These diseases represent the most well-recognized clinical outcomes associated with HTLV-1 infection.

Beyond these common diseases, HTLV-1 has been linked to several inflammatory and immune-mediated conditions-including neurological^5^ and pulmonary diseases^7^ ^),(^ ^8^, ophthalmological manifestations^9^ ^),(^ ^10^, dermatological conditions such as infective dermatitis^11^ ^),(^ ^12^, and rheumatologic disorders-in PLWHTLV-1^13^. Moreover, PLWHTLV-1 may increase susceptibility to certain co-infections, including tuberculosis, which may further complicate clinical outcomes^14^.

Despite the increasing recognition of these complications, the full spectrum of diseases associated with HTLV-1 remains incompletely characterized. Reports describing these manifestations are often distributed across studies on specific organ systems or clinical outcomes. Therefore, a comprehensive synthesis of the available evidence is necessary to better understand the range of pathological conditions associated with HTLV-1. This study offers a systematic review to summarize the main diseases, co-infections, and clinical manifestations in PLWHTLV-1.

## MATERIALS AND METHODS

This systematic review was conducted following a predefined protocol describing its rationale, objectives, and methodological approach. The review followed the recommendations of the Preferred Reporting Items for Systematic Reviews and Meta-Analyses (PRISMA), a checklist designed to support the development of transparent and reproducible systematic review protocols^15^.

### Inclusion criteria

Studies in peer-reviewed journals on the clinical manifestations associated with HTLV-1 were considered eligible. Studies on co-infections, proviral load, and clinical conditions affecting different organ systems (including pulmonary, neurological, dermatological, rheumatological, and ophthalmological diseases) were included in this review.

### Search strategy

A systematic literature search was performed on PubMed, Scopus, Web of Science, and SciELO. The search strategy combined the following keywords: “HTLV-1,” “co-infections,” “opportunistic diseases,” “inflammatory diseases,” “HAM,” “ATL,” “cancers,” and “proviral load.”

### Study selection

**Figure 1:**
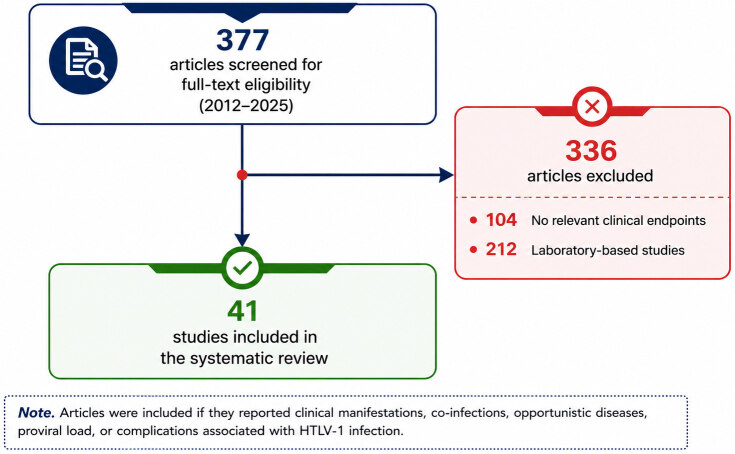
Flowchart summarizing the identification and selection of studies in this systematic review. Articles published from 2012 to 2025 were screened for eligibility. After full-text assessment, studies without relevant clinical endpoints or focused exclusively on laboratory experiments were excluded. A total of 41 articles were included in the final analysis.

Studies were chosen in two stages. First, their titles and abstracts were screened to identify potentially relevant studies. Articles meeting the chosen eligibility criteria were assessed via full-text review. The following relevant information was extracted from the selected studies: authors, year of publication, study design, population characteristics, and main findings (Figure 1).

## RESULTS

The studies in this systematic review associate HTLV-1 infection with a broad and heterogeneous spectrum of clinical manifestations, far beyond the neurological and hematological diseases traditionally linked to the virus^16^. In addition to HTLV-1-associated HAM/TSP and ATL, the reviewed literature showed the substantial involvement of inflammatory, autoimmune, infectious, pulmonary^7^ ^),(^ ^8^ ^),(^ ^14^, dermatological^11^ ^),(^ ^12^ ^),(^ ^17^, ophthalmologica^9^ ^),(^ ^10^, and rheumatological conditions^13^ ^),(^ ^18^ ^-^ ^21^, highlighting the systemic nature of HTLV-1 infection.

A consistent finding across the analyzed studies refers to the central role of chronic immune activation and virus-induced immune dysregulation in the pathogenesis of these manifestations^22^ ^),(^ ^23^. Persistent inflammatory responses associated with HTLV-1 infection seem to contribute to tissue damage and organ-specific inflammatory diseases and to increased susceptibility to opportunistic infections and co-infections^14^ ^),(^ ^22^ ^),(^ ^24^ ^),(^ ^25^. The most frequently reported complications includes tuberculosis^14^ ^),(^ ^24^ ^),(^ ^25^, infective dermatitis^11^ ^),(^ ^12^ ^),(^ ^17^, *Strongyloides stercoralis* infection^26^ ^),(^ ^27^, crusted scabies^26^ ^),(^ ^27^, and several rheumatological manifestations^13^ ^),(^ ^18^ ^-^ ^21^.

To facilitate the clinical interpretation of these findings, this study reorganized manifestations into four major categories according to their predominant pathogenic and clinical characteristics: (a) classical HTLV-1-associated diseases (including HAM/TSP and ATL), (b) other inflammatory and immune-mediated diseases associated with HTLV-1, (c) opportunistic infections and co-infections associated with HTLV-1-related immune dysfunction, and (d) other malignancies potentially associated with chronic HTLV-1 infection.

## CLASSICAL HTLV-1-ASSOCIATED DISEASES

### HTLV-1-associated HAM/TSP

HTLV-1-associated HAM/TSP is a chronic, progressive neurological disorder characterized by spastic paraparesis, bladder dysfunction, and sensory disturbances^5^ ^),(^ ^28^. It shows slowly progressive spastic paraparesis, neurogenic bladder dysfunction, and, to a lesser extent, sensory alterations. Studies highlight that the pathogenesis involves an exacerbated immune response against HTLV-1-infected cells in the central nervous system, particularly in the spinal cord^5^ ^),(^ ^28^. This response leads to demyelination and axonal degeneration, resulting in the typical motor and autonomic symptoms of the disease.

### High proviral load and neurological symptoms

High proviral load is one of the main biomarkers associated with the risk of HAM/TSP. Individuals with elevated proviral load have been reported to present a higher frequency of neurological symptoms (such as hand paresthesia) and increased levels of inflammatory cytokines, including IFN-γ, TNF, and IL-10. However, long-term follow-up studies have shown that, despite this exacerbated inflammatory response, not all persons with high proviral load progress to HAM/TSP (in follow-up periods ranging from three to 16 years), suggesting that factors beyond proviral load and inflammation may contribute to the pathogenesis of the disease^29^.

### ATL

ATL, a rare but aggressive malignancy of mature T-cells, stems from chronic infection with HTLV-1. It typically develops after decades of asymptomatic viral persistence, particularly in individuals infected during childhood via breastfeeding^27^.

### Epidemiology and risk

ATL occurs most prevalently in Japan, the Caribbean, South America, and parts of Africa. Estimates set the lifetime risk of developing ATL among HTLV-1 carriers at 1%-5%, but this risk increases to over 20% in individuals with high proviral load. ATL occurs more often in older adults (60-70 years in Japan), but younger onset has been reported in Latin America and the Caribbean^27^ ^),(^ ^30^.

### Clinical variants

ATL has four clinical subtypes, each with distinct features and prognosis:

- Acute: Rapid progression, systemic symptoms, high lactate dehydrogenase, and circulating malignant cells.
- Lymphomatous: Predominantly lymph node involvement without leukemia.
- Smoldering: Minimal symptoms, often limited to skin or lungs.

### Pathogenesis

HTLV-1 integrates into the host’s genome and promotes monoclonal expansion of infected T-cells. ATL arises from the accumulation of genetic mutations in these clones over time. Premalignant clones can be detected years before clinical onset, offering potential for early intervention^27^.

### HTLV-1-associated uveitis (HU)

HTLV-1 is also implicated in ocular inflammation (particularly uveitis), sicca syndrome (marked by dry eyes and mouth), and interstitial keratitis^9^ ^),(^ ^10^ ^),(^ ^31^. HTLV-1-associated uveitis (HU) is a well-documented inflammatory eye disease caused by HTLV-1 infection^32^. It constitutes one of the most common ocular manifestations of HTLV-1 and can be the only clinical sign of infection in some patients.

### Clinical features

HU typically presents as intermediate or anterior uveitis with either granulomatous or non-granulomatous inflammation. Common symptoms include blurred vision^9^ ^),(^ ^10^, floaters^10^ ^),(^ ^31^, photophobia^10^ ^),(^ ^31^, and mild ocular discomfort^10^ ^),(^ ^31^. It often causes vitreous opacities and retinal vasculitis^9^ ^),(^ ^10^ ^),(^ ^31^. 

### Pathophysiology

The inflammation may be immune-mediated, triggered by HTLV-1-infected T-cells infiltrating ocular tissues. Cytokine production and immune cell activation contribute to the chronic inflammatory response in HU^10^ ^),(^ ^31^.

### HTLV-1-associated infective dermatitis

Infective dermatitis, a recurrent and exudative skin condition, is another notable manifestation in children and adults with HTLV-1^11^ ^),(^ ^12^ ^),(^ ^17^.

HTLV-1-associated infective dermatitis constitutes an inflammatory skin manifestation initially described in children that also affects adults^12^. It causes exudative lesions in areas such as the scalp, face, and intertriginous regions. The prevalence of skin diseases in patients with HTLV-1 can exceed 75%, occurring more often in individuals with HAM^11^ ^),(^ ^17^. Manifestations include xerosis, acquired ichthyosis^33^, dermatophytosis and recurrent bacterial infections^34^, reflecting the chronic immunological and inflammatory dysfunction induced by the virus^11^ ^),(^ ^17^ ^),(^ ^33^.

## OTHER INFLAMMATORY DISEASES ASSOCIATED WITH HTLV-1

### Bronchiectasis and pulmonary disorders

HTLV-1 is associated with chronic inflammatory pulmonary diseases, including bronchiectasis^34^, interstitial pneumonias, bronchiolitis^7^ ^),(^ ^8^ ^),(^ ^36^ ^-^ ^40^, alveolitis^8^ ^),(^ ^37^ ^-^ ^40^, and other interstitial lung diseases^7^ ^),(^ ^8^ ^),(^ ^36^ ^-^ ^40^. These manifestations are particularly frequent in individuals with HAM/TSP. They may be associated with chronic inflammatory infiltration mediated by infected lymphocytes.

### Pulmonary diseases

Bronchiectasis: One of the most frequent pulmonary manifestations in HTLV-1 patients, especially in individuals with HAM/TSP^7^ ^),(^ ^8^ ^),(^ ^36^ ^-^ ^40^. Chronic inflammation may progressively destroy the bronchial architecture, resulting in irreversible bronchial dilation.

Interstitial pneumonias^37^, bronchiolitis^7^ ^),(^ ^8^ ^),(^ ^36^ ^-^ ^40^, alveolitis^8^ ^),(^ ^37^ ^-^ ^40^, and other interstitial lung diseases^7^ ^),(^ ^8^ ^),(^ ^36^ ^-^ ^40^

Risk factors: HAM/TSP, ethnic background, and immunological factors have been associated with a higher risk of bronchiectasis. On the other hand, proviral load has shown no direct correlation with pulmonary lesion severity^7^ ^),(^ ^14^ ^),(^ ^38^. These conditions reflect the diffuse involvement of the lung parenchyma and small airways, with persistent interstitial and alveolar inflammation.

Clinical manifestations: Chronic productive cough, dyspnea, and recurrent respiratory infections are commonly reported. In many cases, symptoms may remain mild or subclinical, making early diagnosis challenging^7^ ^),(^ ^8^ ^),(^ ^36^ ^-^ ^40^.

Risk factors: HAM/TSP, ethnic background, and immunological factors have been associated with a higher risk of bronchiectasis. On the other hand, proviral load has shown no consistently direct correlation with the severity of pulmonary lesions^7^ ^),(^ ^14^ ^),(^ ^38^.

### Clinical and radiological features

Radiological findings: High-resolution computed tomography is essential for diagnosis^41^ ^),(^ ^42^. Findings include:

- Bilateral bronchial dilation (bronchiectasis)
- Bronchial wall thickening
- Pulmonary fibrosis in some cases
- No clear correlation between proviral load and CT findings

### Rheumatologic diseases

Additionally, HTLV-1 infection has been associated with several rheumatological and autoimmune manifestations, including Sjögren’s syndrome, rheumatoid arthritis-like polyarthritis, chronic polyarthritis, interstitial keratitis, polymyositis, and myositis. Sjögren’s syndrome, in particular, affects the exocrine glands and often presents with xerostomia and keratoconjunctivitis sicca^13^ ^),(^ ^18^ ^-^ ^21^.

Persistent activation of T lymphocytes and production of inflammatory cytokines may contribute to the development of articular and autoimmune manifestations^13^ ^),(^ ^18^ ^-^ ^21^. Recent studies suggest that the presence of HTLV-1 may modify the clinical course of these diseases, making them more refractory to conventional treatment.

### Thyroid disorders

Autoimmune thyroid disorders, including Hashimoto’s thyroiditis and Graves’ disease, have also been linked to HTLV-1 infection^4^ ^),(^ ^22^ ^),(^ ^35^.

### Proviral load and disease progression 

### Relationship between proviral load and clinical manifestations

The HTLV-1 proviral load (PVL), defined as the number of viral DNA copies integrated into host cells, is considered one of the main biomarkers associated with disease progression and inflammatory activity in HTLV-1 infection^43^. Elevated PVL has been consistently associated with an increased risk of developing HTLV-1-associated diseases, particularly HAM/TSP and ATL^29^ ^),(^ ^43^.

### Key findings

Longitudinal studies have shown that individuals with persistently high PVL are more likely to develop neurological manifestations, increased inflammatory cytokine production, and clonal expansion of infected T cells^43^. In a large prospective study, none of the individuals with PVL below 4 copies/100 peripheral blood mononuclear cells developed ATL, whereas those with higher PVL showed a markedly increased risk of disease progression^43^.

Although PVL is strongly associated with inflammatory and neoplastic manifestations, not all individuals with elevated proviral load develop clinical disease, suggesting that additional host, immunological, and viral factors may contribute to disease susceptibility and progression^27^ ^),(^ ^29^ ^),(^ ^43^.

### Infectious and autoimmune diseases associated with HTLV-1

Analysis of the included studies showed that HTLV-1-associated HAM/TSP was the most frequently reported HTLV-1-associated disease, followed by HU and HTLV-1-associated infective dermatitis. These conditions represent an important source of morbidity in PLWHTLV-1.

The relatively high frequency of autoimmune manifestations, such as Sjögren’s syndrome and inflammatory arthritis, further highlights the complex immune dysregulation associated with HTLV-1 infection (Table 1).

**Table 1:** Frequency of clinical manifestations associated with HTLV-1

Type of disease	Frequency	Percentage (%)
HTLV-1-associated myelopathy	20/41	48.8%
Uveitis	12/41	29.3%
Infective dermatitis	9/41	22.0%
Sjögren’s syndrome	8/41	19.5%
Arthritis (rheumatoid and psoriatic)	9/41	22.0%
Bronchiectasis	10/41	24.4%
Graves’ disease	4/41	9.8%
Xerophthalmia	2/41	4.9%
Xerostomia	2/41	4.9%
Interstitial keratitis	2/41	4.9%
Polymyositis / Myositis	3/41	7.3%

Frequency of diseases associated with HTLV-1 reported in the studies included in this review (n = 41). The table shows the number and percentage of studies describing each clinical manifestation.

## OPPORTUNISTIC INFECTIONS ASSOCIATED WITH HTLV-1

### Tuberculosis

In summary, HTLV-1 infection presents a complex clinical profile involving widespread inflammatory pathologies. Awareness and early identification of these conditions are essential for improving patient outcomes^26^.

Recent studies reinforce the association between HTLV-1 and tuberculosis, with a prevalence of HTLV-1 in up to 10% of patients with pulmonary tuberculosis in Brazil^24^. HTLV-1 infection compromises the T-cell-mediated immune response, essential for the control of *Mycobacterium tuberculosis* ^26^, which may explain the higher incidence and severity of tuberculosis in these patients. Furthermore, co-infected individuals have a higher risk of extrapulmonary forms and unfavorable clinical evolution^14^ ^),(^ ^25^ ^),(^ ^44^.

Our results also indicate that tuberculosis is the most frequently HTLV-1-associated opportunistic disease, followed by *Strongyloides stercoralis* and crusted scabies. These diseases represent a significant clinical challenge for PLWHTLV-1 due to the immune dysregulation associated with the virus. The high prevalence of tuberculosis highlights the need for close monitoring and appropriate management in this population (Table 2).

**Table 2:** Frequency of opportunistic infections in PLWHTLV-1

Type of disease	Frequency	Percentage (%)
Tuberculosis	18/41	43.9%
*Strongyloides stercoralis* infection	7/41	17.1%
Crusted scabies	2/41	4.9%
*Mycobacterium leprae* infection	2/41	4.9%
*Schistosoma mansoni* infection	2/41	4.9%
Community-acquired pneumonia	1/41	2.4%
*Molluscum contagiosum*	1/41	2.4%
Cytomegalovirus infection	1/41	2.4%
Herpes virus infection	1/41	2.4%
Toxoplasma infection	1/41	2.4%

Frequency and percentage of studies on opportunistic infections in PLWHTLV-1 (n = 41). Tuberculosis was the most prevalent opportunistic infection, followed by *Strongyloides stercoralis* and crusted scabies, reflecting the immunosuppressive impact of HTLV-1 and underscoring the need for vigilant monitoring and management.

### Strongyloidiasis

*Strongyloides stercoralis* infection has been consistently reported as the second most frequently opportunistic infection associated with HTLV-1 (following tuberculosis). It represents an important complication related to virus-induced immune dysregulation. Co-infection with HTLV-1 is associated with impaired host immune responses against helminths, particularly due to alterations in Th2-mediated immunity and reduced production of cytokines involved in eosinophil activation and parasite clearance^26^ ^),(^ ^27^.

Thus, individuals co-infected with HTLV-1 and *Strongyloides stercoralis* may present more severe clinical manifestations, including recurrent infection, hyperinfection syndrome, and disseminated strongyloidiasis, conditions associated with increased morbidity and mortality^27^. Moreover, some studies suggest that chronic strongyloidiasis may contribute to persistent immune activation and potentially influence HTLV-1 proviral load and disease progression.

The association between HTLV-1 and strongyloidiasis is particularly relevant in endemic regions (in which both infections frequently overlap), reinforcing the importance of early diagnosis and appropriate antiparasitic treatment in people living with HTLV-1^45^.

### Crusted scabies

Crusted scabies has also been described as the third most frequent opportunistic infection associated with HTLV-1, following tuberculosis^23^ ^),(^ ^26^ ^),(^ ^27^. This virus causes a range of clinical manifestations, from inflammatory conditions, including neuronal damage (HTLV-1 associated myelopathy, HAM^.^ This severe form of scabies includes extensive hyperkeratotic skin lesions, high parasite burden, and increased transmissibility, particularly in individuals with impaired immune responses^27^.

The association between HTLV-1 and crusted scabies is believed to be related to virus-induced immune dysregulation, especially altered cell-mediated immunity^22^ ^),(^ ^23^ ^),(^ ^26^ ^),(^ ^27^. Individuals co-infected with HTLV-1 may present recurrent or more severe infestations, with increased risk of secondary bacterial infections and systemic complications^27^.

Because crusted scabies may reflect significant immunological dysfunction, its recognition in people living with HTLV-1 is clinically important, particularly in endemic regions in which both conditions may coexist^23^ ^),(^ ^27^.

## OTHER CANCERS POTENTIALLY ASSOCIATED WITH HTLV-1 INFECTION

This review shows that, as expected, ATL is the cancer most frequently associated with HTLV-1 (46.3% of cases). However, other malignancies, including liver, lung, gastric, cervical, skin, spleen, and gastrointestinal cancers, also occur, albeit less frequently, highlighting that HTLV-1-associated oncogenesis goes beyond ATL. Nonetheless, the predominance of ATL underscores the critical need for continued research into the molecular mechanisms underlying HTLV-1-driven cancer and the development of effective therapeutic strategies^22^ ^),(^ ^27^ ^),(^ ^30^ ^),(^ ^46^ (Table 3).

**Table 3:** Frequency of cancers associated with HTLV-1

Type of cancer	Frequency	Percentage (%)
ATL	19/41	46.3%
Liver	2/41	4.9%
Lung	2/41	4.9%
Gastric	1/41	2.4%
Cervical	1/41	2.4%
Skin	1/41	2.4%
Spleen	1/41	2.4%
Gastrointestinal	1/41	2.4%

Frequency and percentage of cancers in PLWHTLV (n = 41). ATL was the most prevalent malignancy, whereas other cancers (including liver, lung, gastric, cervical, skin, spleen, and gastrointestinal) occurred less often, highlighting that HTLV‑1‑associated oncogenesis goes beyond ATL.

Our review shows that ATL remains the main malignancy associated with HTLV-1 infection. Nevertheless, additional malignancies involving the liver, lung, stomach, cervix, skin, spleen, and gastrointestinal tract also emerged in the reviewed literature.

Table 4 summarizes the articles in this review.

## DISCUSSION

One of the most consistent observations in the reviewed studies is the strong association between HTLV-1 infection and chronic inflammatory disorders affecting multiple organ systems. Neurological manifestations (particularly HAM/TSP) remain the most extensively studied complications of HTLV-1 infection. The pathogenesis of HAM/TSP is believed to involve an exaggerated immune response against HTLV-1-infected T cells infiltrating the central nervous system, leading to chronic spinal cord inflammation and progressive neurodegeneration^5^ ^),(^ ^28^. In line with previous studies, the summarized results indicate that proviral load plays an important role in disease risk as individuals with higher proviral loads tend to show stronger inflammatory responses and a higher likelihood of neurological manifestations^29^ ^),(^ ^43^.

However, an important and still unresolved issue concerns the incomplete predictive value of proviral load for disease progression. Although elevated proviral load is strongly associated with HAM/TSP and ATL, longitudinal studies have shown that many persons with persistently high proviral loads remain asymptomatic for long periods^29^. This observation suggests that additional host and viral factors, such as genetic background, immune regulation, viral integration patterns, and clonal expansion dynamics, may significantly influence disease outcomes^27^. Thus, proviral load alone may fail to suffice as a prognostic biomarker, highlighting the need for more comprehensive predictive models incorporating immunological and genetic factors.

**Table 4:** Included studies on clinical manifestations and complications associated with HTLV-1

Article	Short title	Year	Location	Study type
Yamano and Sato^5^	Clinical pathophysiology of HTLV-1-associated myelopathy/tropical spastic paraparesis	2012	Kawasaki, Japan	Review
Einsiedel *et al.* ^7^	Proviral load, lung disease, and survival in HTLV-1 subtype C infection	2018	Australia	Prospective cohort
Dias *et al.* ^8^	An overview of human T-lymphotropic virus type 1 lung injury	2018	Para, Brazil	Mini-review
Kamoi *et al.* ^9^	Horizontal transmission of HTLV-1 causing uveitis	2021	Tokyo, Japan	Case report
Ozores *et al.* ^10^	Prevalence of HTLV-1-associated uveitis in Bahia	2023	Bahia, Brazil	Cross-sectional
Bittencourt and Farre^11^	Infective dermatitis in HTLV-1: underdiagnosed disease	2024	Bahia, Brazil	Review
Garcia *et al*.^12^	Infective dermatitis mimicking atopic dermatitis	2022	Rio Grande do Sul, Brazil	Case report
Umekita^13^	HTLV-1 and rheumatoid arthritis clinical course	2022	Japan	Review
Souza *et al.* ^14^	Tuberculosis status, neurologic disease, and immune response in HTLV-1	2017	Bahia, Brazil	Retrospective
Silva *et al.* ^16^	Drug candidates targeting HTLV-1	2023	Para, Brazil	Review
Oliveira *et al.* ^17^	Infective dermatitis associated with HTLV-1: evaluation of 42 cases in Bahia	2015	Bahia, Brazil	Observational
Umekita and Okayama^18^	HTLV-1 infection and rheumatic diseases	2020	Miyazaki, Japan	Mini-review
Tsukimata *et al*.^19^	Rheumatological manifestations in HTLV-1/2	2025	Para, Brazil	Cross-sectional
Nakamura *et al.* ^20^	HTLV-1 and Sjögren’s syndrome phenotypes	2022	Tokyo, Japan	Review
Nakamura *et al.* ^21^	Clinical manifestations in HTLV-1-seropositive patients with Sjögren’s syndrome	2015	Nagasaki, Japan	Retrospective
Martin *et al.* ^22^	Inflammatory manifestations of HTLV-1 and therapeutic options	2014	London, UK	Review
Schierhout *et al.* ^23^	HTLV-1 infection and adverse health outcomes: systematic review and meta-analysis	2020	Australia	Systematic review and meta-analysis
Keikha and Karbalaei^24^	HTLV-1 and tuberculosis coinfection	2021	Mashhad, Iran	Mini-review
Grassi *et al.* ^25^	Tuberculosis incidence in HTLV-1-infected individuals	2016	Bahia, Brazil	Retrospective cohort
Rosadas and Taylor^26^	HTLV-1 and coinfections	2022	London, UK	Review
Legrand *et al.* ^27^	Public health implications of HTLV-1 infection	2022	Australia	Review
Nakamura^28^	HAM/TSP pathogenesis and T-cell transmigration	2023	Nagasaki, Japan	Review
Ferraz *et al.* ^29^	Clinical and immunologic features in HTLV-1 carriers with high proviral load	2020	Bahia, Brazil	Prospective cohort
Dahy *et al.* ^30^	Lung carcinoma in HTLV-1 infection: case report and review	2021	Sao Paulo, Brazil	Case report
Kamoi *et al.* ^31^	Updates on HTLV-1 uveitis	2022	Tokyo, Japan	Review
Kamoi and Mochizuki^32^	HTLV-1 uveitis	2012	Tokyo, Japan	Mini-review
McGill *et al.* ^33^	HTLV-1-associated infective dermatitis: updates on pathogenesis	2012	Cardiff, UK	Review
Souza *et al.* ^34^	Infective dermatitis in adults with HTLV-1: clinicopathological aspects	2020	Bahia, Brazil	Retrospective
Honarbakhsh and Taylor^34^	Bronchiectasis linked to HTLV-1-associated inflammatory disease	2015	London, UK	Retrospective
Einsiedel *et al.* ^36^	Predictors of non-cystic fibrosis bronchiectasis in Indigenous adult residents of central Australia: results of a case-control study	2019	Australia	Case-control
Einsiedel *et al.* ^37^	HTLV-1-associated pulmonary disease: clinical features	2021	Australia	Review
Einsiedel *et al*.^38^	HTLV-1 subtype C proviral load associated with bronchiectasis in Indigenous Australians	2014	Australia	Prospective cohort
Normando *et al.* ^39^	HTLV-I induces lesions in the pulmonary system: A systematic review	2020	Para, Brazil	Systematic review
Dias *et al.* ^40^	Human T lymphotropic virus and pulmonary diseases	2018	Para, Brazil	Review
Kimura *et al.* ^41^	Bronchioloalveolar disorder in HTLV-1: case report	2024	Japan	Case report
Cachay *et al.* ^42^	Pulmonary disease in HTLV-1 patients without tuberculosis	2021	Lima, Peru	Cross-sectional
Taylor *et al.* ^43^	Proviral load predicting HTLV-1-associated disease	2024	London, UK	Review
Morales and Montero^44^	HTLV-1 and tuberculosis coinfection (fatal case)	2024	Colombia	Case report
Ye *et al.* ^45^	Human T-cell lymphotropic virus type 1 and *Strongyloides stercoralis* co-infection: a systematic review and meta-analysis	2022	London, UK	Systematic review and meta-analysis
Wang *et al.* ^46^	Current therapeutics for HTLV-1	2024	Australia	Review
Phillips^47^	Treatment advances in ATLL	2023	New York, USA	Review

Another key finding emerging from the reviewed literature is the strong association between HTLV-1 infection and increased susceptibility to opportunistic infections, particularly tuberculosis. Several epidemiological studies have shown higher rates of tuberculosis in PLWHTLV-1, especially in endemic regions such as Brazil^14^ ^),(^ ^24^ ^),(^ ^25^. This association is likely related to HTLV-1-induced alterations in cell-mediated immunity, particularly dysfunctional CD4+ T-cell responses, which are essential for controlling *Mycobacterium tuberculosis* ^26^. In addition to increased susceptibility, co-infected individuals seem to experience more severe disease and a higher frequency of extrapulmonary manifestations^14^ ^),(^ ^25^ ^),(^ ^44^. These findings emphasize the importance of systematic screening for tuberculosis in PLWHTLV-1, particularly in high-burden settings.

Dermatological manifestations also represent a prominent component of HTLV-1-associated morbidity. HTLV-1-associated infective dermatitis has long been recognized as an important clinical marker of infection, particularly in pediatric populations^12^ ^),(^ ^17^. More recent studies, however, have shown that similar inflammatory skin conditions may also occur in adults^34^. In addition to HTLV-1-associated infective dermatitis, several other dermatological disorders (including xerosis, acquired ichthyosis, and recurrent bacterial or fungal infections) have been reported with high frequency in PLWHTLV-1^11^ ^),(^ ^17^ ^),(^ ^33^. These manifestations likely reflect chronic immune activation and impaired skin barrier function, further supporting the concept that HTLV-1 induces persistent systemic immune dysregulation.

Rheumatological and autoimmune manifestations represent another important and somewhat controversial aspect of HTLV-1 infection. Several studies have reported associations between HTLV-1 and autoimmune conditions such as Sjögren’s syndrome, rheumatoid arthritis-like polyarthritis, and other inflammatory arthropathies^13^ ^),(^ ^18^ ^-^ ^21^. The mechanisms underlying these associations remain incompletely understood but may involve chronic activation of infected T cells and dysregulated cytokine production^18^. Interestingly, some reports suggest that HTLV-1 infection may alter the clinical course of autoimmune diseases, potentially leading to more refractory disease or modified therapeutic responses^12^ ^),(^ ^13^. Nevertheless, the causal relationship between HTLV-1 and certain autoimmune disorders remains debated as it is often difficult to distinguish direct viral effects from coincidental coexistence in endemic populations.

Pulmonary disease associated with HTLV-1 infection has received increasing attention in recent years, but remains underrecognized in clinical practice. Bronchiectasis seem to be one of the most frequent pulmonary complications, particularly in individuals with HAM/TSP^35^ ^-^ ^38^. Chronic inflammatory infiltration of the airways by HTLV-1-infected lymphocytes may contribute to progressive structural damage and bronchial dilation^8^ ^),(^ ^39^. Interestingly, several studies have reported that pulmonary disease severity does not consistently correlate with proviral load^7^ ^),(^ ^38^, suggesting that local immune responses and tissue-specific inflammatory processes may play a more prominent role than systemic viral burden in the pathogenesis of pulmonary complications.

Ophthalmological manifestations, particularly HU, represent another well-established complication of HTLV-1 infection. HU causes the inflammatory infiltration of ocular tissues by HTLV-1-infected lymphocytes and can present as anterior or intermediate uveitis^9^ ^),(^ ^31^. In some patients, ocular inflammation may even represent the first clinical manifestation of HTLV-1 infection^31^. Notably, HU has been reported in persons with HAM/TSP and in otherwise asymptomatic carriers, suggesting that ocular involvement may occur independently of neurological disease^31^. This highlights the importance of ophthalmological evaluations in individuals diagnosed with HTLV-1 infection.

As expected, ATL remains the most severe and life-threatening HTLV-1-associated disease in this review. ATL develops after decades of persistent infection, resulting from the clonal expansion of infected T cells, which progressively accumulate oncogenic mutations^27^. Although the lifetime risk of ATL among HTLV-1 carriers is relatively low (approximately 1%-5%), the prognosis of aggressive forms remains poor despite advances in treatment^27^ ^),(^ ^47^. Interestingly, this review also found reports of other malignancies in PLWHTLV-1. While these cancers occur much less frequently than ATL, their presence raises important questions about whether HTLV-1-induced immune dysregulation may contribute to broader oncogenic susceptibility^27^ ^),(^ ^30^. However, current evidence remains insufficient to establish a direct causal relationship between HTLV-1 and most non-ATL malignancies.

## CONCLUSION

Overall, the results of this systematic review highlight several important gaps in the current knowledge. First, the true prevalence of many HTLV-1-associated inflammatory conditions remains uncertain, largely due to underdiagnosis and limited epidemiological surveillance in endemic regions^23^. Second, although proviral load widely serves as a marker of disease risk, its predictive value remains incomplete, indicating the need for additional biomarkers to find persons at the highest risk of disease progression^43^. Third, the literature has many cross-sectional studies, limiting the ability to determine causal relationships between HTLV-1 infection and specific clinical manifestations.

These findings emphasize the need for a broader clinical perspective when managing PLWHTLV-1. Rather than solely focusing on ATL and HAM/TSP, clinicians should be aware of the diverse inflammatory, infectious, autoimmune, and pulmonary complications associated with the virus. Early recognition and multidisciplinary monitoring may be essential to improving long-term outcomes for this population.

## Data Availability

The complete anonymized dataset supporting the findings of this study is included within the article itself.
